# Deficits in explicit emotion regulation in bipolar disorder: a systematic review

**DOI:** 10.1186/s40345-021-00221-9

**Published:** 2021-05-03

**Authors:** Marcel Kurtz, Pia Mohring, Katharina Förster, Michael Bauer, Philipp Kanske

**Affiliations:** 1 Clinical Psychology and Behavioral Neuroscience, Faculty of Psychology, Technische Universität Dresden, Chemnitzer Str. 46, 01187 Dresden, Germany; 2 Department of Psychiatry and Psychotherapy, Medical Faculty, University Hospital Carl Gustav Carus, Technische Universität Dresden, Dresden, Germany; 3 Max Planck Institute for Human Cognitive and Brain Sciences, Leipzig, Germany

**Keywords:** Explicit emotion regulation, Bipolar disorder, Systematic review, Frontostriatal network, Reappraisal

## Abstract

**Background:**

This study aimed to compile and synthesize studies investigating explicit emotion regulation in patients with bipolar disorder and individuals at risk of developing bipolar disorder. The importance of explicit emotion regulation arises from its potential role as a marker for bipolar disorders in individuals at risk and its potent role in therapy for bipolar disorder patients.

**Methods:**

To obtain an exhaustive compilation of studies dealing specifically with explicit emotion regulation in bipolar disorder, we conducted a systematic literature search in four databases. In the 15 studies we included in our review, the emotion-regulation strategies *maintenance*, *distraction*, and *reappraisal* (self-focused and situation-focused) were investigated partly on a purely behavioral level and partly in conjunction with neural measures. The samples used in the identified studies included individuals at increased risk of bipolar disorder, patients with current affective episodes, and patients with euthymic mood state.

**Results:**

In summary, the reviewed studies' results indicate impairments in explicit emotion regulation in individuals at risk for bipolar disorder, patients with manic and depressive episodes, and euthymic patients. These deficits manifest in subjective behavioral measures as well as in neural aberrations. Further, our review reveals a discrepancy between behavioral and neural findings regarding explicit emotion regulation in individuals at risk for bipolar disorders and euthymic patients. While these groups often do not differ significantly in behavioral measures from healthy and low-risk individuals, neural differences are mainly found in frontostriatal networks.

**Conclusion:**

We conclude that these neural aberrations are a potentially sensitive measure of the probability of occurrence and recurrence of symptoms of bipolar disorders and that strengthening this frontostriatal route is a potentially protective measure for individuals at risk and patients who have bipolar disorders.

## Background

The primary characteristics of bipolar disorders (BD) are aberrations of mood and emotion (American Psychiatric Association 2013; Gruber 2011; Phillips 2003; Townsend and Altshuler 2012). These aberrations manifest in episodes of euphoric and excessively elevated mood (i.e., manic and hypomanic episodes), with manic episodes indicating type 1 bipolar disorder (BD-I) and hypomanic episodes indicating type 2 (BD-II). Typically, episodes of excessive negative affect (i.e., depressive episodes) accompany both types of BD. Further, patients with BD exhibit aberrations in the processing of emotional stimuli (Mercer and Becerra 2013; Rosen and Rich 2010; Townsend and Altshuler 2012; Wessa et al. 2014; Wessa and Linke 2009), which are at least partly due to deficits in the regulation of emotions (Aldao et al. 2010; Dodd et al. 2019; Green et al. 2011; Gruber et al. 2013b; Phillips 2003; Phillips et al. 2008; Townsend and Altshuler, 2012). Deviations in patients with BD occur in everyday, spontaneous, and automatic ER (i.e., implicit ER) and explicit ER, which describes conscious, planned processes. Explicit ER is trainable and can be volitionally integrated into everyday life and is therefore of particular interest because of its high potential as a treatment target in therapeutic intervention. Because of this potential, our aim in the present article is to review and integrate studies that investigate explicit ER in BD. Furthermore, as recent studies addressed the neural correlates of such explicit ER, we also aim to summarize this literature to conclude implications for the treatment of BD and identify open questions regarding the neural processes underlying different ER strategies.

### Rational

ER denotes processes that enable us to modulate the experience and the expression of our emotions (Gross 1998b). Strategies of ER have been classified, for instance, according to their explicitness (Phillips et al. 2008), motivational aspects (Koole, 2009), or the chronological order of regulation (Gross 1998a, 1998b, 2013, 2015).

#### Antecedent-focused and response-focused emotion regulation

The influential classification of Gross (1998a) distinguishes two categories, depending on whether the regulation of emotions takes place in anticipation of, or response to, emotions.

Antecedent-focused regulation strategies involve selecting and modifying the situation in which we experience emotions and modify emotions via cognition (Gross 1998a, 2013; Phillips et al. 2008). Cognitive processes can be further differentiated into subprocesses on a continuum between pure attentional processes and processes of cognitive change (Ochsner and Gross, 2005). An example of an attentional strategy is *distraction,* which describes the detachment of attention from a stimulus that evokes emotions to other, non-emotional aspects of the situation, away from the situation entirely, or non-emotional thoughts (Gross, 2013). In contrast to simple attentional processes, cognitive change includes higher cognitive abilities that lead to an alternative evaluation of a situation that evokes emotions. Cognitive change can either be caused by concentrating on one’s coping strategies or an alternative interpretation of the situation (i.e., reappraisal). Reappraisal can be further distinguished in self-focused and situation-focused reappraisal (Ochsner et al. 2004). While self-focused reappraisal describes the modification of the degree of personal involvement in an emotionally loaded situation, situation-focused reappraisal describes the reinterpretation of the situation itself.

Response-focused strategies include the suppression or alteration of behavioral, experiential, and physiological responses that accompany emotions. They further include processes that enable us to prolong or maintain the experience of emotions (Gross 1998b; Tugade and Fredrickson 2007). To accomplish emotion maintenance, an individual has to express the behavioral response associated with an emotion (Izard 1990), for example, yelling in case of anger. Further, an individual can maintain an emotion in a working-memory system specialized for emotions (Mikels et al. 2008). Notably, the listed ER strategies are not valence specific; both negative and positive emotions can be regulated.

#### Neural basis of emotion regulation

Neurally, emotion regulation is accomplished through the interaction of two systems. (Ochsner and Gross 2014). One system is responsible for the experience and the contextual valuation of emotions, consisting of the amygdala, insula, ventral striatum, and medial areas of the prefrontal cortex (PFC). A control system acts on this system, consisting of the dorsal anterior cingulate cortex (dACC), dorsal posterior medial PFC, dorsolateral PFC (DLPFC), inferior parietal cortex, and ventrolateral prefrontal cortex (VLPFC). In part, specific cognitive processes are associated with specific areas within this network. For example, the DLPFC, the dorsal posterior mPFC, and the inferior parietal cortex are associated with the control of attention to relevant stimuli and the maintenance of goals during emotion regulation. The ventrolateral PFC is associated with inhibiting inappropriate behaviors, and the dACC is associated with monitoring conflicts between desirable and actual actions. For the involvement of most of the mentioned areas, meta-analytical evidence has been accumulated (Kohn et al. 2014). While the model of Ochsner and Gross (2014) is characterized by a high level of detail, including the attribution of cognitive processes to specific areas, it does not distinguish between explicit and implicit ER, concepts whose importance to ER more recently has been demonstrated and which are the focus of the next section.

#### Implicit and explicit emotion regulation

In recent research, ER strategies have been classified according to how voluntarily they occur (Braunstein et al. 2017; Gross, 2013; Gyurak et al. 2011; Phillips et al. 2008). While implicit ER runs spontaneously, automatically, and unconsciously, explicit ER operates planned, consciously, and volitionally. A further distinction can be made between whether the goal of the ER is explicit or implicit and how automatic or controlled the ER process itself is (Braunstein et al. 2017). In the present review, we adhere to a strict definition of explicit ER in which both the goal of ER is explicit (which is ensured in experimental setups by instruction), and the required processes are controlled.

**Fig. 1:**
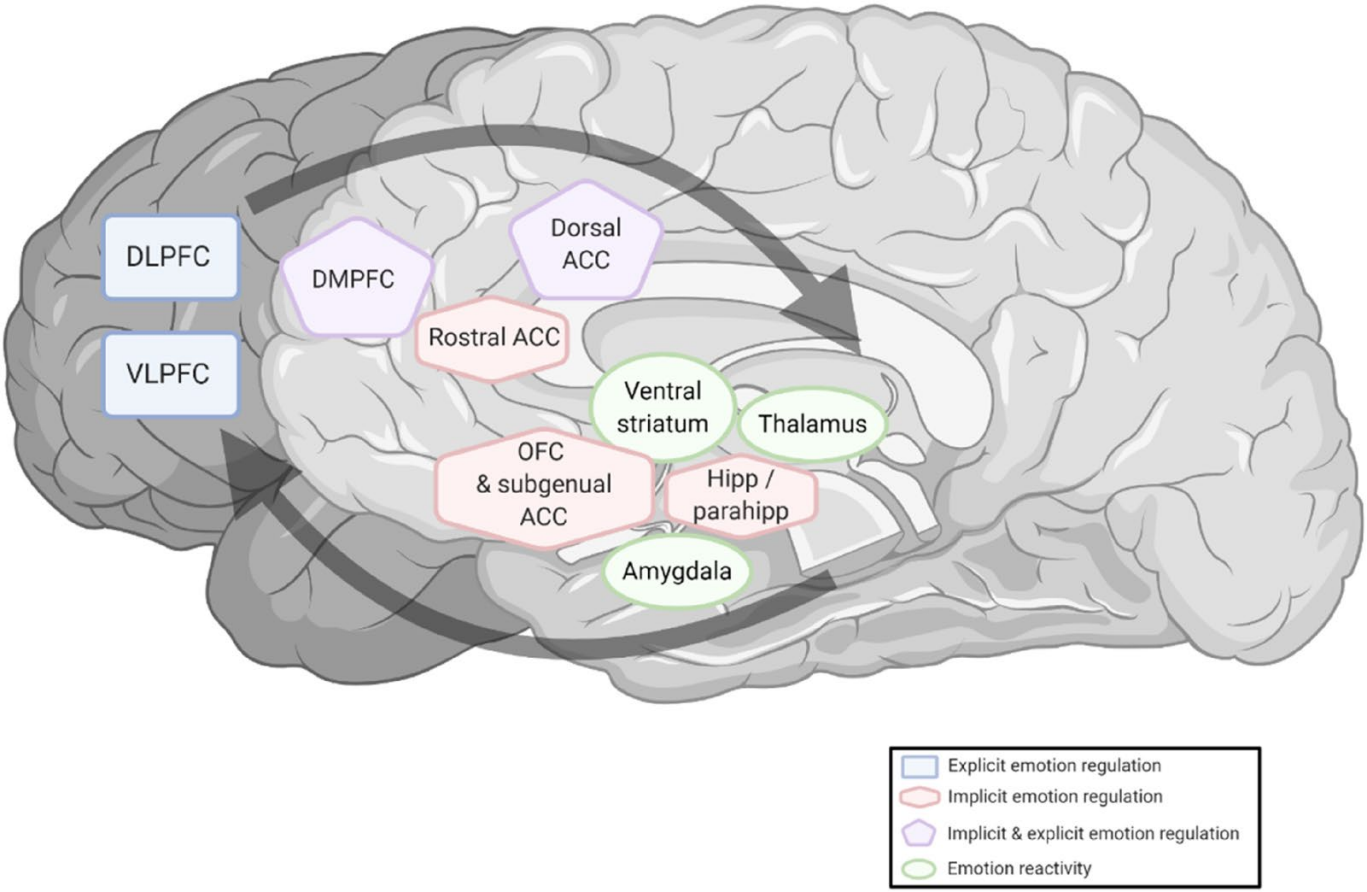
Simplified neural model of emotion regulation. Illustration of areas involved in emotion reactivity, implicit emotion regulation, and explicit emotion regulation. Black arrows indicate feedforward and feedback mechanisms of these subsystems. Adapted from “A neural model of voluntary and automatic emotion regulation: implications for understanding the pathophysiology and neurodevelopment of bipolar disorder” by Phillips et al. (2008). Created with BioRender.com

Explicit and implicit ER are further distinguishable by their demands on different neural networks. The neural model by Phillips et al. (2008) postulates a hierarchical organization of implicit and explicit ER (Fig. 1). On the lowest level, areas involved in emotion reactivity (i.e., ventral striatum, thalamus, and amygdala) are embedded in a network, together with areas involved in automatic ER (i.e., rostral anterior cingular cortex (rACC), subgenual anterior cingular cortex, orbitofrontal cortex (OFC), and a hippocampus-parahippocampus region). On the highest level, explicit ER is primarily realized by prefrontal areas (dorsolateral prefrontal cortex (DLPFC) and the ventrolateral prefrontal cortex (VLPFC)) but mediated by areas associated with implicit ER (such as the OFC) that, in turn, have direct connections to subcortical areas associated with emotion reactivity. According to the model, the dorsomedial prefrontal cortex (DMPFC) and dorsal anterior cingulate cortex fulfill functions in explicit and implicit ER.

In addition to the consideration of explicit and implicit ER, the model of Phillips et al. (2008) has the advantage that it includes findings on the ER of patients with bipolar disorders. Based on previous results (Elliott et al. 2004; Wessa et al. 2007), the researchers concluded that inefficient use of the DLPFC and VLPFC results in increased activity in these regions and is, thus, a probable cause for deficit regarding explicit ER in patients with BD. However, due to the model's hierarchical structure, aberrations in areas associated with implicit ER or emotion reactivity could also impair explicit ER. For this reason, an alternative explanation is that hyperactivity in DLPFC and VLPFC may represent a compensatory mechanism for aberrations in areas associated with implicit ER and emotion reactivity.

Taken together, the model of Phillips et al. (2008) provides essential insights into the neural basis of ER deficits in patients with BD. However, the model is based on only a few studies, especially for explicit ER, which leads to limitations since the results are derived from homogeneous samples and low diversity tasks.

Patients who have BD show aberrations not only at the neural level but also in the experience of emotions and associated behavior. Studies sampling experiences in everyday life found an increased frequency of attempts to regulate emotions in BD patients (Gruber et al. 2013b). However, these spontaneous emotion-regulation attempts are less successful compared to healthy subjects (Gruber et al. 2012), which is partly due to the more frequent use of maladaptive strategies (e.g., rumination) by patients with BD (Becerra et al. 2013; Dodd et al. 2019; Green et al. 2011; Gruber et al. 2012; Gruber et al. 2013b). Interestingly, in laboratory settings, differences between BD patients and healthy controls (HC) are less pronounced (Dodd et al. 2019). A possible explanation for this phenomenon could be that participants in laboratory settings are often explicitly instructed to use ER; that is, they initiate ER voluntarily, which could positively affect their success. This positive effect makes explicit ER strategies an exciting topic for research as explicit ER strategies might be a resource to build on in psychotherapeutic interventions.

### Objectives

Contrary to implicit processes, which are difficult to influence by an individual, explicit ER strategies allow for the volitional integration into everyday life. Most importantly, it has been pointed out that implicit ER processes can be altered via extensive training of explicit ER (Denny and Ochsner 2014; Gyurak et al. 2011). Due to training, explicit ER may then become habitual, implicit, and automatic. This hypothesis is supported by findings that suggest an overlap of the neural circuits underlying explicit and implicit ER (Phillips et al. 2008). In summary, due to their trainability, explicit ER strategies are of particular interest in the development of therapeutic approaches and interventions related to ER in BD.

Recent studies also stress the importance of early detection of BD risk in individuals for early intervention and prophylactic treatment (Malhi et al. 2017; Almeida and Phillips 2013; Phillips and Kupfer 2013). For this topic, studies investigating explicit ER in risk groups could be particularly revealing since risk groups may have ER aberrations even before the development of BD, making explicit ER a potential marker for early detection of the disorder. In contrast to this trait marker hypothesis (Rohde et al. 1990), the scar hypothesis (Rohde et al. 1990) claims that impairments in ER of BD patients are more likely a consequence of an experienced affective episode. If alterations in explicit ER already appear in risk groups, another interesting question is how specific these aberrations are for BD, given that these aberrations are also characteristic of other mental disorders. For example, aberrations in ER also occur in schizophrenia (Khoury and Lecomte 2012) and patients with depression (Rive et al. 2013). Due to depression and BD's similarity in their characterization by affective episodes, the distinction between these two disorders is particularly challenging. Recently it has been shown that unipolar patients can be distinguished from bipolar patients based on valence-specific aberrations in emotion reactivity (Bürger et al. 2017; Surguladze et al. 2010; Grotegerd et al. 2013; Lawrence et al. 2004). Accordingly, the question arises whether individuals with BD risk also show valence-specific aberrations specific for BD.

In the present review, we aim to provide an overview of what behavioral and neural aberrations are present in individuals at risk for BD, patients with current affective episodes, and euthymic patients during explicit ER. From this, we aim to conclude the suitability of explicit ER as a potential marker for BD or as a marker for relapse into affective episodes. Furthermore, we aim to draw implications for the use of ER in the treatment of BD.

## Methods

To obtain an exhaustive compilation of studies addressing specifically explicit ER in BD, we conducted a systematic literature search. We used a systematic approach to identify relevant papers in the Medline, PsycINFO, PubMed, and Web of Science databases. The search was conducted in December 2018. A criterion regarding the publication date was not included. According to our criteria, all abstracts of the identified papers were read and included or excluded for the further review process. Our search included all studies in which the title or abstract contained the words Bipolar Disorder, Bipolar Affective Disorder, or Bipolar Mood Disorder, together with the words Emotion Regulation, Affect Regulation, Emotion Control, or Affect Control. The search term was ("Bipolar Disorder" OR "Bipolar Affective Disorder" OR "Bipolar Mood Disorder") AND ("Emotion Regulation" OR "Affect Regulation" OR "Emotion Control" OR “Affect Control”). Our search revealed 487 studies. To these studies, we applied further inclusion criteria. First, we exclusively included studies in which explicit ER was instructively induced. This criterion is consistent with the strict definition of explicit ER that we base the review on because instruction ensures that ER goals are deliberate and planned, and the specific processes take place in a controlled manner. The second inclusion criterion was that the studies used subjects with a diagnosed BD and/or a proven propensity and/or genetic vulnerability corresponding to these disorders. Fifteen studies met the inclusion criteria. The full text of all included studies was inspected.

## Results

**Fig. 2:**
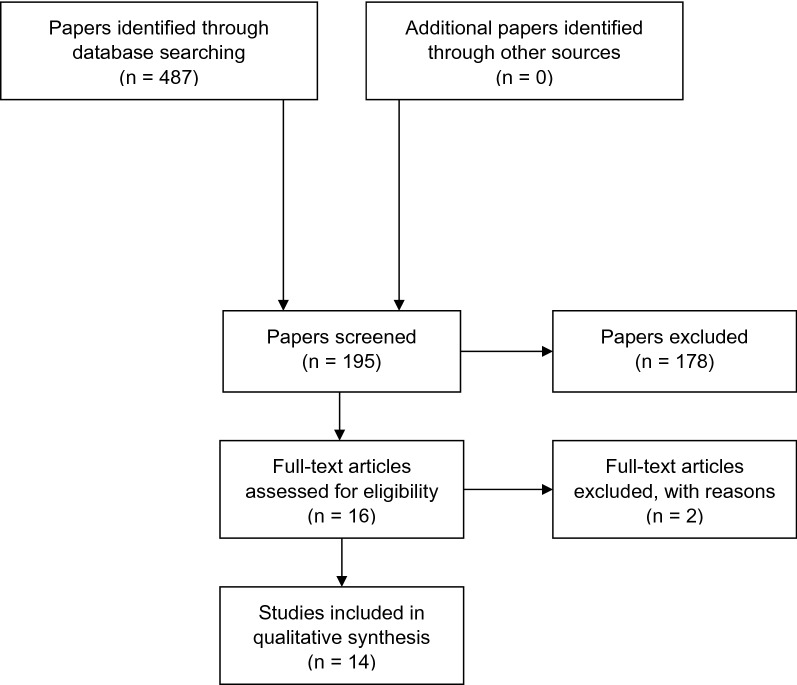
Prisma flow diagram of the selection of papers

Studies we identified in our literature search investigated the ER strategies maintenance, distraction, and reappraisal (self-focused as well as situation-focused). Figure 2 shows the selection steps of the literature search. The results of these studies are summarized in the section below. We grouped the studies depending on ER strategies. Within the sections on strategies, we have further subdivided the studies according to which the patient or risk group was investigated. As both terms, *euthymic* and *remitted*, describe patients who have been diagnosed with BD but who did not have a manic, depressive or mixed mood at the time of the study, we will not further differentiate between these states.

### Maintenance of emotions

Only one study investigated processes concerning the maintenance of emotions in BD (Gruber et al. 2013a). The results are shown in Table 1. Maintenance of emotion refers to sustaining an emotion that contributes to goal-oriented behavior beyond the presence of the emotion-triggering stimulus. In this study, the authors tested subjects with BD-I in euthymic mood state and HC in an emotional working memory task (Larkin and Cartensen 2005; Mikels et al. 2008). Participants received the instruction to maintain an emotion evoked by a picture during a delay phase. At the end of the delay phase, participants saw a second picture and compared the two pictures' emotional intensity. Afterward, participants rated each picture regarding emotional intensity. This study's dependent variable represents the degree of concordance between the comparison task and the subsequent ratings. Euthymic BD-I patients did not differ from HC in the maintenance of positive emotions. However, patients with BD-I were less able to maintain negative emotions than HC. In sum, the study revealed deficits in maintaining negative emotions in euthymic patients with BD-I.

**Table 1:** Studies that investigated the maintenance of emotions in euthymic BD patients

Study	Age	Medication	Paradigm	Dependent variables	Results behavioral	Results neural
Gruber et al. (2013a, b)	Adults	Yes	Task: Emotional working memory task Valence: Negative, positive Participants: BD-I, remitted (n = 29) HC (n = 30)	Behavioral: Performance in the emotion working memory task	Maintaining negative emotions: BD-I < HC Maintaining positive emotions: NS	X

*BD* bipolar disorder, *HC* healthy controls, UD, *NS* not significant

### Regulating emotions via distraction

Six studies that we identified investigated distraction as ER strategy in BD (Caseras et al. 2015; Heissler et al. 2014; Kanske et al. 2013, 2015; Ladouceur et al. 2013; Lois et al. 2017). An overview of the findings on distraction in risk groups can be found in Table 2, Table 3 shows an overview of the findings on distraction in euthymic patients.

**Table 2:** Studies that investigated distraction in risk groups

Study	Age	Medication	Paradigm	Dependent variables	Results behavioral	Results neural
Ladouceur et al. (2013)	Adolescents	No	Task: N-back task with emotional distractors Valence: Negative, positive Participants: HBO (n = 15) HC (n = 16)	Behavioral: Performance in n-back task Neural: fMRI, PPI Regions of interest: Amygdala, Ventral Striatum, ventrolateral prefrontal cortex (VLPFC), DLPFC, and anterior cingulate cortex (ACC) PPI seed: VLPFC PPI targets: Amygdala, DLPFC	Distraction from emotional distractors: NS	During distraction from emotional distractors: Right VLPFC: happy: Risk group > HC Right VLPFC: fearful: NS PPI during distraction from emotional distractors: VLPFC – Amygdala (positive connectivity) Fearful: HC > Risk group (right amygdala) Happy: HC > Risk group (left amygdala) Neutral: HC vs. Risk group: NS Right VLPFC – left DLPFC (positive connectivity) Fearful: HC vs. Risk group: NS Happy: HC > Risk group Neutral: HC vs. Risk group: NS
Kanske et al. (2013)	Adults	No	Task: Picture viewing task Valence: Negative, positive Participants: High HPS scores (n = 22) FDR (n = 17) HC (n = 22, n = 17)	Behavioral: Performance in the distraction task Neural: fMRI	RT (mental arithmetic task): NS	During distraction: HC vs. Risk groups: NS
Heissler et al. (2014)	Young adults	No	Task: Picture viewing task Valence: Negative, positive Participants: High HPS scores (n = 22) HC (n = 24)	Behavioral: Self-report (during and after the task) Neural: fMRI Regions of interest: Emotions reactivity: Amygdala, subgenual ACC, VMPFC, occipital and ventral temporal cortices, thalamus Emotion regulation: OFC, DLPFC, DMPFC, dACC, parietal cortex, precuneus	Rating after the experiment: Risk group > HC Viewing condition: NS Emotion regulation: NS Performance in the mental arithmetic task: NS	Viewing: Negative vs. neutral: Right Amygdala: Risk group > HC Positive vs. neutral: NS Distraction vs. viewing: Negative: NS Positive: left inferior parietal cortex: Risk group > HC
Kanske et al. (2015)	Adults	No	Task: Picture viewing task Valence: Negative, positive Participants: FDR (n = 17) HC (n = 17)	Behavioral: Self-report during and after the task Neural: fMRI Regions of interest: Bilateral OFC, dorsolateral (DLPFC, middle frontal) and dorsomedial prefrontal (DMPFC, superior medial), anterior cingulate (ACC), and parietal cortex (inferior, superior)	Rating after the experiment: Risk group > HC (all pictures more positive) Viewing: Positive: Risk group < HC Negative: NS Distraction: NS	Viewing: NS Distraction vs. viewing: NS

*ACC* anterior cingulate cortex, *dACC* dorsal anterior cingulate cortex, *DLPFC* dorsolateral prefrontal cortex, *DMPFC* dorsomedial prefrontal cortex, *fMRI* functional magnetic resonance imaging, *FDR* first-degree relatives, *HBO* healthy bipolar offspring, *HC* healthy controls, *HPS* hypomanic personality scale, *NS* not significant, *OFC* orbitofrontal cortex, *PFC* prefrontal cortex, *PPI* psychophysiological interaction, *VLPFC* ventrolateral prefrontal cortex, *VMPFC* ventromedial prefrontal cortex

**Table 3:** Studies that investigated distraction in euthymic BD patients

Study	Age	Medication	Paradigm	Dependent variables	Results behavioral	Results neural
Caseras et al. (2015)	Adults	Yes	Task: N-back task with emotional distractors Valence: Negative, positive Participants: BD-I, euthymic (n = 16) BD-II, euthymic (n = 19) HC (n = 20)	Behavioral: Performance in n-back task Neural: fMRI, PPI, DTI Regions of interest: DLPFC, amygdala, and accumbens PPI seed: DLPFC PPI targets: Amygdala, accumbens	RT (2-back vs. 0-back): BD-I > BD-II and HC Slowing due to distractors in 2-back task: BD-I > BD-II and HC RT in 2-back without distractors: BD-I > HC BD-II vs. HC: NS RT in the 2-back task (no distractor vs. emotional distractor): BD-I: Fear > no distractor Happy > no distractor BD-II: NS HC: Fear > no distractor Neutral > no distractor	2-back (no distractor) vs. 0-back (no distractor): Working memory network: BD-I > BD-II and HC 2-back task (distractor vs. no distractor): DLPFC: Fear: BD-II > BD-I > HC Happy: BD-I > BD-II and HC Neutral: HC > BD-I Amygdala: Fear: BD-II > BD-I > HC Happy: BD-I > BD-II > HC Neutral: BD-I > HC Accumbens: Fear: BD-I and BD-II > HC Happy: BD-I > BD-II and HC Neutral: BD-I > HC Functional connectivity: DLPFC-Amygdala Fear: BD-II > BD-I and HC DLPFC-Accumbens NS Structural integrity: Left uncinate fasciculus: NS Right uncinate fasciculus: BD-I < BD-II and HC
Kanske et al. (2013)	Adults	Yes	Task: Picture viewing task Valence: Negative, positive Participants: BD-I, euthymic (n = 22) HC (n = 22)	Behavioral: Performance in the distraction task Neural: fMRI	RT in the mental arithmetic task: BD-I > HC	During distraction: Right parietal cortex: BD-I > HC
Kanske et al. (2015)	Adults	Yes	Task: Picture viewing task Valence: Negative, positive Participants: BD-I, euthymic (n = 22) HC (n = 22)	Behavioral: Self-report during and after the task Neural: fMRI Regions of interest: Bilateral OFC, dorsolateral (DLPFC, middle frontal) and dorsomedial prefrontal (DMPFC, superior medial), anterior cingulate (ACC), and parietal cortex (inferior, superior)	Self-report: Rating after the experiment: NS Viewing: Positive: NS Negative: NS Distraction: NS	Viewing: NS Distraction vs. viewing: NS

*ACC* anterior cingulate cortex, *BD* bipolar disorder, *DLPFC* dorsolateral prefrontal cortex, *DMPFC* dorsomedial prefrontal cortex, *DTI* diffusion tensor imaging; fMRI, functional magnetic resonance imaging, *HC* healthy controls, *NS* not significant, *OFC* orbitofrontal cortex, *PFC* prefrontal cortex, *PPI* psychophysiological interaction, *VLPFC* ventrolateral prefrontal cortex, *VMPFC* ventromedial prefrontal cortex

Note, Heissler et al. (2014) and Kanske et al. (2015) also investigated reappraisal as an ER strategy in addition to distraction. In this section, we only address their results regarding distraction. Lois et al. (2017) explicitly compared reappraisal and distraction as ER strategies, which we, therefore, review in another corresponding section. Tasks used to measure distraction have in common that they contain a cognitive task and emotional stimuli presentation. Interestingly, the studies investigating distraction in BD can be distinguished by their independent variables: while some studies focus on the self-ratings of emotion and associated neural activity as independent variables (Heissler et al. 2014; Kanske et al. 2015), others focus on the influence of emotion on the performance in cognitive tasks and associated neural activity as independent variables (Kanske et al. 2013; Ladouceur et al. 2013). For the study of attentional ER in BD, both measures are of interest since both cognitive task losses and increased emotional ratings indicate impaired distraction from emotional content. In the following section, we grouped studies according to their task rather than their independent variables.

#### Distraction in people at risk for BD

Ladouceur et al. (2013) used an emotional n-back task to investigate alterations of voluntary, attentional ER and its neural correlates. In this task’s 0-back condition, the attention demand is low (a simple keystroke is requested for a specific stimulus). In the 2-back condition, the demand is high (a keystroke is required when a stimulus is the same as in the second last trial). In some of the task’s trials, task-irrelevant pictures, either emotional or neutral faces, are presented. Participants show the ER by controlling their attention to focus on the n-back task and ignoring the emotional distractors. Therefore, a heightened error rate and an extended response time (RT) in the n-back task during the presence of emotional distractors indicate failures in ER.

Interestingly, the authors chose to investigate healthy offspring of patients with BD compared to healthy low-risk controls (i.e., offspring of parents, neither of whom had been diagnosed with an axis 1 disorder). The n-back task performance in healthy offspring of BD patients and low-risk controls did not differ significantly. However, in the high demanding 2-back condition, both groups differed in neural activity evoked by emotional distractors. BD patients’ healthy offspring showed hyperactivity in the VLPFC in the presence of happy but not fearful distractors. In the presence of both types of emotional distractors, the healthy offspring of BD patients showed a reduced modulation of the amygdala by the VLPFC. Additionally, in the presence of happy distractors, healthy offspring of BD patients showed a reduced modulation of the DLPFC by the VLPFC.

Heissler et al. (2014) examined individuals with an increased risk for BD (indicated by high scores in the Hypomanic Personality Scale (HPS; (Eckblad and Chapman 1986) and compared their performance in a picture viewing task (positive, negative, and neutral stimuli) with a low-risk group. In the picture viewing task, participants look at pictures with emotional content (Heissler et al. 2014; Kanske et al. 2013, 2015). They receive either the instruction to simply view the pictures or to regulate their emotions. In this particular version of the task, participants could distract themselves from emotions by solving an arithmetical task presented in addition to the pictures.

During the processing of emotions in the passive viewing condition, the risk group exhibited increased amygdala activity in response to negative stimuli compared to the control group. Behaviorally, the risk group showed no significant impairments in ER. However, participants with increased risk for BD exhibited enhanced amygdala activity in the passive viewing condition and increased activity in the left inferior parietal cortex in the distraction condition.

Kanske et al. (2015) used the same setup. Individuals at risk for BD did not significantly differ from HC concerning behavioral and neural measures during distraction. Kanske et al. (2013) used a similar setup and partly the same sample as Kanske et al. (2015), examining two risk groups. For the first, an increased risk was indicated by high HPS scores. The second risk group consisted of unaffected first-degree relatives of patients with BD. This study investigated the activity of the neural network to emotional stimuli during the arithmetic task. No significant losses or aberrations concerning neural measures were observed in the risk groups.

In sum, there was no evidence on the behavioral level that people at risk for BD differ significantly from HC in distracting themselves from negative or positive emotions (Heissler et al. 2014; Kanske et al. 2013, 2015; Ladouceur et al. 2013). Concerning neural measures, patients at risk for BD showed increased neural activity while distracting themselves from positive emotional stimuli in the VLPFC (Ladouceur et al. 2013) and the inferior parietal cortex (Heissler et al. 2014). Despite increased activity in the VLPFC, connectivity analyses revealed a lower modulation of the amygdala (for positive and negative stimuli) and the DLPFC (for positive stimuli) by the VLPFC in the risk group (Ladouceur et al. 2013) during distraction. These findings contrast with studies that found no significant differences regarding neural measures between people at risk for BD and HC (Kanske et al. 2013, 2015).

#### Distraction in euthymic patients

Caseras et al. (2015) investigated differences in distraction in euthymic patients with BD-I, euthymic patients with BD-II, and HC assessed in an emotional n-back task. Particularly patients with BD-I exhibited impairments. First, this group showed inferior performance in the 2-back condition. Additionally, in the 2-back condition, BD-I patients showed slower RT due to distractors than the other groups. In contrast to the other groups, especially the distraction from happy distractors was impaired. Regarding the neural correlates of ER, patients with BD-I compared to other groups exhibited increased activity in the DLPFC, amygdala, and accumbens when happy distractors were present. In the presence of fear distractors, activity in these areas was highest in patients with BD-II. BD-II patients also showed a higher (inverse) functional connectivity between DLPFC and amygdala when fearful distractors were present. For patients with BD-I, results indicate lower structural integrity in the uncinate fasciculus, a white matter association tract that connects frontal regions with subcortical regions. In contrast, no significant structural aberrations were found for patients with BD-II.

Euthymic patients with BD-I in Kanske et al. (2015) did not significantly differ from HC regarding self-reports of emotions or neural activity during distraction. However, in Kanske et al. (2013), the same patient group solved the arithmetic task slower than HC when emotional pictures were present. In the presence of emotionally background pictures, patients with BD-I, compared to HC, exhibited increased activity in the right parietal cortex.

Regarding distraction in patients with BD, it is essential to note that all patients examined in the cited studies were in a euthymic state. While the self-report of emotions in euthymic BD patients provides no evidence of significant impairment (Kanske et al. 2015), the cognitive measures show the opposite (Kanske et al. 2013; Caseras et al. 2015). Regarding neural measures, patients with BD-I exhibited hyperactivity in DLPFC, amygdala, and nucleus accumbens during distraction from happy pictures and aberrations in structural integrity between frontal areas and the amygdala (Caseras et al. 2015). In contrast, patients with BD-II exhibited heightened activity in these areas and increased inverse functional connectivity between DLPFC and amygdala during the distraction from fearful stimuli (Caseras et al. 2015). Other results indicate no such aberrations in areas associated with emotion reactivity and regulation, but instead aberrations in areas associated with the cognitive distraction task (Kanske et al. 2013).

### Regulating emotions via reappraisal

Our search identified three studies investigating reappraisal abilities of patients with BD without using imaging methods (Ajaya et al. 2016; Gruber et al. 2014; Kjærstad et al. 2016). Seven studies investigated neural correlates in addition to behavioral measures (Corbalán et al. 2015; Heissler et al. 2014; Kanske et al. 2015; Morris et al. 2012; Rive et al. 2015; Townsend et al. 2013; Zhang et al. 2018). The majority of studies that examined reappraisal used a picture viewing task to evoke emotions. Exceptions are Gruber et al. (2014) and Ajaya et al. (2016). Gruber et al. (2014) presented emotional movie clips to participants and instructed them to carefully view the clips or reappraise to regulate their emotions. In the study of Ajaya et al. (2016), a manipulation of the keyboard with which participants navigated in a video game was supposed to induce anger. Three studies investigated reappraisal in participants at risk for BD [indicated by HPS scores (Ajaya et al. 2016; Heissler et al. 2014) and genetic predisposition (Kanske et al. 2015)]. Table 4 shows the results of these studies. Two studies included samples with a current affective episode (Table 5). Patients in Morris et al. (2012) consisted partly of euthymic individuals and partly of hypomanic individuals. Rive et al. (2015) examined patients with BD in a euthymic and depressive state. In seven studies, patients were in a euthymic state (Corbalán et al. 2015; Gruber et al. 2014; Kanske et al. 2015; Kjærstad et al. 2016; Rive et al. 2015; Townsend et al. 2013; Zhang et al. 2018). An overview of these studies is given in Table 6.

**Table 4:** Studies that investigated reappraisal in risk groups

Study	Age	Medication	Paradigm	Dependent variables	Results behavioral	Results neural
Heissler et al. (2014)	Young adults	No	Task: Picture viewing task Valence: Negative, positive Participants: High HPS scores (n = 22) HC (n = 24)	Behavioral: Self-report during and after the task Neural: fMRI Regions of interest: Emotions reactivity: Amygdala, subgenual ACC, VMPFC, occipital and ventral temporal cortices, thalamus; Emotion regulation: OFC, DLPFC, DMPFC, dACC, parietal cortex, precuneus	Rating after the experiment: Arousal: Risk group > HC Viewing condition: NS Reappraisal: NS	Viewing: Negative vs. neutral: Right Amygdala: Risk group > HC Positive vs. neutral: NS Reappraisal vs. viewing: Negative: Right Amygdala: Risk group > HC Positive: NS
Kanske et al. (2015)	Adults	No	Task: Picture viewing task Valence: Negative, positive Participants: FDR (n = 17) HC (n = 17)	Behavioral: Self-report during and after the task Neural: fMRI, PPI Regions of interest: Bilateral OFC, dorsolateral (DLPFC, middle frontal) and dorsomedial prefrontal (DMPFC, superior medial), anterior cingulate (ACC), and parietal cortex (inferior, superior) PPI seed: Amygdala PPI targets: OFC, dorsolateral (DLPFC, middle frontal) and dorsomedial prefrontal (DMPFC, superior medial), anterior cingulate (ACC) and parietal cortex (inferior, superior)	Rating after the experiment: Risk group > HC (all pictures more positive) Viewing: Positive: Risk group < HC Negative: NS Reappraisal: Risk group < HC	Viewing: NS Reappraisal vs. viewing: Amygdala: Risk group > HC PPI: Left amygdala/bilateral OFC: HC, negative connectivity; Risk group, positive connectivity Right amygdala/right OFC: HC, negative connectivity; Risk group, positive connectivity
Ajaya et al. (2016)	Young adults	No	Task: Videogame task Valence: Negative Participants: Undergraduates (n = 66)	Behavioral: self-reported affect, facial expressions Psychophysiology: Respiratory sinus arrhythmia (RSA)	Higher HPS scores increased RSA during ER (only in the deliberated reappraisal condition)	X

*ACC* anterior cingulate cortex, *dACC* dorsal anterior cingulate DLPFC, dorsolateral prefrontal cortex, *DMPFC* dorsomedial prefrontal cortex, *fMRI* functional magnetic resonance imaging, *HC* healthy controls, *HPS* hypomanic personality scale, *OFC* orbitofrontal cortex, *PFC* prefrontal cortex, *PPI* psychophysiological interaction, *VLPFC* ventrolateral prefrontal cortex, *VMPFC* ventromedial prefrontal cortex

**Table 5:** Studies that investigated reappraisal in patients with a current affective episode

Study	Age	Medication use	Paradigm	Dependent variables	Results behavioral	Results neural
Rive et al. (2015)	Adults	No	Task: Picture viewing task Valence: Negative, positive Participants: BD-I/BD-II, currently depressed (n = 9) BD-I/BD-II, remitted (n = 26) HC (n = 36)	Behavioral: Self-report Neural: fMRI Regions of interest: Amygdala, thalamus, insula, DLPFC, ACC, medial PFC, and hippocampus	ER success in depressed groups: Happy: UDd < HC BDd vs. HC: n.s. differences Happy vs. sad: BDd: happy > sad HC: happy vs. sad: NS	Depressive state: Reappraisal of happiness vs. reappraisal of sadness: rACC: BDd > UDd
Morris et al. (2012)	Adults	Yes	Task: Picture viewing task Valence: Negative Participants: BD-I (n = 13) (six BD patients met criteria for euthymia and five met criteria for hypomania) HC (n = 15)	Behavioral: Self-report Neural: fMRI Regions of interest: Amygdala, cortico-lymbic areas with activity during ER Coupling seed: Amygdala Coupling targets: Whole brain	Self-report of emotions: HC: Increase > Maintain > Decrease BD: Increase > Maintain BD: Maintain v.s. Decrease: NS	Down regulation: Right VLPFC: BD > HC Up regulation: Right VLPFC: BD > HC Rostral ACC: BD > HC Inverse coupling of left PFC (especially left IFG) and amygdala found in HC did not occur in BD

*ACC* anterior cingulate cortex, *BD* bipolar disorder, *DLPFC* dorsolateral prefrontal cortex, *fMRI* functional magnetic resonance imaging, *HC* healthy controls, *PFC* prefrontal cortex, *PPI* psychophysiological interaction, *rACC* rostral anterior cingulate cortex

**Table 6:** Studies that investigated reappraisal in euthymic BD patients

Study	Age	Medication use	Paradigm	Dependent variables	Results behavioral	Results neural
Kanske et al. (2015)	Adults	Yes	Task: Picture viewing task Valence: Negative, positive Participants: BD-I, euthymic (n = 22) HC (N = 22)	Behavioral: Self-report during and after the task Neural: fMRI, PPI Regions of interest: Bilateral OFC, dorsolateral (DLPFC, middle frontal) and dorsomedial prefrontal (DMPFC, superior medial), anterior cingulate (ACC), and parietal cortex (inferior, superior) PPI seed: Amygdala PPI targets: OFC, dorsolateral (DLPFC, middle frontal) and dorsomedial prefrontal (DMPFC, superior medial), anterior cingulate (ACC) and parietal cortex (inferior, superior)	Rating after the experiment: NS Viewing: NS Reappraisal: NS	Viewing: NS Reappraisal vs. viewing: Amygdala: BD-I > HC Parahippocampal: BD-I > HC PPI during reappraisal: Left amygdala/right OFC HC negative connectivity, BD-I positive connectivity Left amygdala/ventral ACC: HC negative connectivity, BD-I positive connectivity Right amygdala/right OFC HC negative connectivity, BD-I positive connectivity
Corbalán et al. (2015)	Adults	Yes	Task: Picture viewing task Valence: Negative Participants: BD-I, euthymic (n = 19) HC (n = 17)	Behavioral: Self-report Behavioral fMRI Regions of interest: Whole brain	NS	Viewing negative: VLPFC: BD-I > HC Reappraisal negative: VLPFC: BD-I > HC Reappraisal negative vs. viewing negative: Amygdala: BD-I > HC
Townsend et al. (2013)	Adults	Yes	Task: Picture viewing task Valence: Negative Participants: BD-I, euthymic (n = 30) HC (n = 26)	Neural: fMRI Regions of interest: Bilateral amygdala PPI seed: Bilateral amygdala PPI target: Whole brain (and a priori VLPFC)	No affect related behavioral data was reported	Viewing negative vs. viewing neutral: HC vs. BD-I: NS Reappraisal Negative vs. Viewing Negative: Amygdala: HC vs. BD-I: n.s. differences Bilateral VLPFC: HC > BD-I Insula: HC > BD-I Bilateral MFG: HC > BD-I Bilateral cingulate: HC > BD-I pre-SMA: HC > BD-I PPI Seed: Left amygdala Negative Functional connectivity Left VLPFC: HC > BD-I Left occipital gyrus: HC > BD-I Right posterior cingulate: HC > BD-I PPI Seed: Right amygdala: Negative functional connectivity Right MFG: BD-I > HC
Zhang et al. (2018)	Adults	Yes	Task: Picture viewing task Valence: Negative Participants: BD patients,euthymic (n = 23), (2 currently depressed) HC (n = 17)	Behavioral: Self-report Neural: fMRI, DCM VOI: DLPFC, left VLPFC, and left amygdala	ER and emotion reactivity: NS	GLM contrasts: NS DCM: DLPFC to Amygdala during reappraisal: BD < HC
Gruber et al. (2014)	Adults	Yes	Task: Film clips Valence: Negative, positive Participants: BD-I, remitted (n = 23) HC (n = 23)	Behavioral: Self-report, analysis of facial expression Physiological measures: Skin conductivity and Respiratory sinus arrhythmia	Uninstructed vs. Reappraisal: NS	X
Kjærstad et al. (2016)	Adults	Yes	Tasks: Social scenarios task, Picture viewing task Valence: Negative Participants: BD-I, full or partial remitted (n = 9) BD-II, full or partial remitted (n = 11) Depression, full or partial remitted (n = 20) HC (n = 20)	Behavioral: Self-report	Emotion reactivity: NS Social Scenarios/dampen: Negative: BD < HC Positive: NS Picture task/reappraisal: NS	X
Rive et al. (2015)	Adults	No	Task: Picture viewing task Valence: Negative, positive Participants: BD-I/BD-II, remitted (n = 26) HC (n = 36)	Behavioral: Self-report Neural: fMRI Regions of interest: Amygdala, thalamus, insula, DLPFC, ACC, medial PFC, and hippocampus	ER success in remitted groups across all emotions: BDr < HC	Regulate vs. viewing across emotions: DLPFC: NS

*ACC* anterior cingulate cortex, *BD* bipolar disorder, *DCM* dynamic causal modeling, *DLPFC* dorsolateral prefrontal cortex, *DMPFC* dorsomedial prefrontal cortex, *fMRI* functional magnetic resonance imaging, *HC* healthy controls, *HPS* hypomanic personality scale, *OFC* orbitofrontal cortex, *PFC* prefrontal cortex, *PPI* psychophysiological interaction, *VLPFC* ventrolateral prefrontal cortex

#### Reappraisal in individuals at risk for BD

Heissler et al. (2014) did not find significant differences in behavioral measures of reappraisal in HC and participants at risk for BD. However, individuals at risk for BD exhibited hyperactivity in the amygdala during the reappraisal of negative emotions, indicating deficits in reappraisal, which did not manifest in behavioral measures.

Kanske et al. (2015) were able to clarify the role of frontal areas in the reappraisal of participants at risk for BD and euthymic patients with BD-I using the same setup as Heissler et al. (2014). In contrast to Heissler et al. (2014), differences in ER abilities were found for the high-risk group compared to their controls, with the high-risk group showing a decreased downregulation of positive emotions during reappraisal. Regarding neural measures, the high-risk group exhibited increased activity of the amygdala during reappraisal compared to HC. Most importantly, further analyses revealed that this was due to an impaired downregulation of the amygdala by OFC in the reappraisal condition.

Like Heissler et al. (2014), Ajaya et al. (2016) measured the risk for BD with the HPS. The authors were particularly interested in the effect of explicit and implicit instruction on reappraisal in individuals at risk for BD. Subjects in the video game task either received the instruction to concentrate on the game, were implicitly primed with the sentence-unscrambling task (Mauss et al. 2007) to apply reappraisal, or were explicitly instructed to apply reappraisal. Increased expression of anger indicated aberrant emotion reactivity in individuals with higher HPS scores. Interestingly, in Ajaya et al. (2016), HPS scores were correlated with respiratory sinus arrhythmia (RSA) deviations from baseline during ER after explicit instruction. This effect was absent when the instruction was implicitly primed or when participants received no instruction. Because RSA is an ER indicator, the authors conclude that these results suggest that explicit instruction enabled participants with higher HPS scores to successfully control their emotions. A characteristic of this analog study that has to bear in mind is the sample whose members were undergraduates and on a wide range of HPS scores. Thus, it differs from the strict definition of risk groups in the other studies reviewed in this section.

Taken together, the results of studies investigating reappraisal in participants at risk for BD revealed aberrations in emotion reactivity as well as in the ability to reappraise emotions. Regarding reappraisal, aberrations were found on behavioral (Kanske et al. 2015), as well as on neural levels (Heissler et al. 2014; Kanske et al. 2015). Neurally, aberrant emotion reactivity of negative emotions is reflected by hyperactivity in the amygdala (Heissler et al. 2014; Kanske et al. 2015). In participants at risk for BD, amygdala hyperactivity was associated with aberrant connectivity between the OFC and the amygdala during reappraisal (Kanske et al. 2015). While in HC, both areas were inversely coupled, they were positively associated with the risk group. The studies provide equivocal findings regarding the extent to which neural aberrations also affect the behavioral level. In two of three studies investigating reappraisal in people at risk for BD, no significant impairments at the behavioral level were found (Ajaya et al. 2016; Heissler et al. 2014).

#### Reappraisal in patients with current manic and depressive episodes

Rive et al. (2015) investigated ER in a mixed sample, which included patients who had BD-I, patients who had BD-II, and HC in a picture viewing task with negative and positive stimuli in an fMRI setting. Patient groups were further divided into two subgroups depending on whether they were currently remitted (BDr) or depressed (BDd). In this section, we review the results of BD patients with a depressive episode; the results regarding remitted patients are reviewed in a corresponding section below. A notable characteristic of this study is that exclusively self-focused reappraisal was used. In healthy individuals, self-focused reappraisal has been shown to be less effective than situation-focused reappraisal (Willroth and Hilimire 2016). However, because situation-focused reappraisal is a relatively complex cognitive process, it has been argued that self-focused reappraisal for patients with affective disorders is more comfortable to apply (Rive et al. 2015). For BD-patients in a depressed state, the reappraisal of sad emotions compared to the reappraisal of happy emotions was compromised. Neurally, this patient group showed a decreased activity in rACC during the regulation of happy and hyperactivity during the regulation of sad emotions.

A distinguishing feature of Morris et al. (2012) is the BD-patient sample, which includes euthymic patients and patients who currently suffered from hypomanic episodes. Subjects were instructed to either use situation- or self-focused reappraisal, intensify, or maintain their emotions in a picture viewing task. The subjective affect rating revealed no significant differences between the patients and the group across all conditions. However, while the control group reported lower affect after the decrease condition than the maintain condition, affect ratings of patients who suffered from BD did not differ significantly between these conditions. Regarding the upregulation of emotions, both groups were able to increase their emotions compared to the maintenance of emotions. At the neural level, BD patients, as compared to HC, showed aberrant hyperactivity in the right VLPFC during up- and downregulation. Additionally, hyperactivity in rACC was found in BD patients during upregulation. Patients who suffered from BD differed from HC in the functional connectivity between areas in the frontal cortex and the amygdala. A negative correlation between activity in the left IFG and activity in the amygdala, found in HC during the downregulation of emotions, was absent in BD patients.

The studies investigating reappraisal in patients with current affective episodes show an affect-congruent impairment of reappraisal in the depressed state associated with hypoactivity in the rACC. Further, the opposite pattern was found in patients with a hypomanic episode. Here, affect-incongruent deviations in reappraisal were shown. These were associated with hyperactivity in the rACC and aberrations in fronto-limbic connectivity. A different approach to investigate reappraisal in BD was taken in the following studies. In these studies, reappraisal in patients with euthymic mood state was investigated.

#### Reappraisal in euthymic patients

In four studies we identified in our literature search, reappraisal abilities of patients with BD at a euthymic state were investigated (Corbalán et al. 2015; Kanske et al. 2015; Townsend et al. 2013; Zhang et al. 2018). Three further studies described the patient group's mood state as remitted (Gruber et al. 2014; Kjærstad et al. 2016; Rive et al. 2015).

In the study of Gruber et al. (2014), euthymic subjects that suffered from BD-I and HC saw movie clips that induced neutral, positive, or negative emotions. Instructions were to either observe these clips or to use reappraisal to modify emotions. Emotions were assessed by self-report, facial expression, and physiological measures (skin conductivity and respiratory sinus arrhythmia). Both groups were successful in reappraisal, implying intact reappraisal in euthymic patients.

Kjærstad et al. (2016) investigated reappraisal in patients with BD (a mixed group that contained BD-I patients and patients with BD-II) and HC. Patients were in a partly or fully remitted state. Two different ER paradigms were used in this study. First, in a social scenario task, participants read fictional social scenarios and corresponding self-beliefs. The scenarios could either be positive, negative, or neutral. Participants were instructed to either typically react to the scenarios or to dampen their emotions. Second, participants applied reappraisal in a picture viewing task. The authors assumed that the social scenarios task evokes stronger emotions due to patients' higher personal involvement. Indeed, in the social scenarios task, patients with BD were less able to downregulate negative emotions than HC.

In contrast, in the picture viewing task, patients with BD did not significantly differ from HC in reappraising negative emotions. It is difficult to conclude the divergent findings in both tasks since both tasks differ not only in the stimulus material but also in the instruction. The instruction to dampen emotions in the social scenarios task may be more challenging to implement than clearly defined reappraisal. Nevertheless, like Gruber et al. (2014), Kjærstad et al. (2016) did not found indications for significant deficits in reappraisal in euthymic patients with BD. However, it is not clear from either study whether euthymic patients and HC differ in their neural activity regarding reappraisal. Fortunately, the following studies addressed this question.

Euthymic patients in Rive et al. (2015) showed impaired self-focused reappraisal of happy and sad emotions. Neurally, patients with BD in a euthymic state did not significantly differ from HC during self-focused reappraisal.

Regarding neural measures, euthymic patients with BD-I in Kanske et al. (2015) showed amygdala hyperactivity during reappraisal compared to HC. Further analyses revealed aberrations in functional connectivity between the amygdala and the OFC, and the ventral ACC. While in HC, the connection between these areas was reverse, in euthymic patients, the connection was positive.

Corbalán et al. (2015) investigated differences in the reappraisal of euthymic patients with BD-I and HC during a picture viewing task. Self-report of emotions indicated that euthymic BD-I patients and HC were able to regulate their emotions successfully. Regarding self-report measurement, tests between the two groups did not reveal significant differences. However, both groups differed concerning neural activity. BD-I patients showed no reduction in amygdala activity during the reappraisal of negative emotions that healthy control participants exhibited. Further, in contrast to HC, patients with BD-I showed an involvement of the VLPFC in the neutral reappraisal condition and the negative viewing condition.

While Corbalán et al. (2015) give insights into neural activity during reappraisal, Townsend et al. (2013) primarily focused on differences in functional connectivity between the amygdala and frontal regions in euthymic patients with BD-I. This group showed reduced activity in bilateral VLPFC, insula, bilateral middle frontal gyrus (MFG), cingulate, and pre-supplementary motor area (SMA). Most importantly, reduced negative connectivity between the left amygdala and left VLPFC, left occipital gyrus, and right posterior cingulate was found in the patient group during reappraisal. In contrast, negative connectivity between the right amygdala and the right MFG was enhanced in BD-I patients. The results of Townsend et al. (2013) do not allow any conclusions about the causal direction of aberrant connectivity between the amygdala and other regions in patients with BD. This causal direction was addressed by Zhang et al. (2018).

Zhang et al. (2018) investigated causal connectivity between frontal areas (namely DLPFC and VLPFC) and the amygdala. Participants in this study were euthymic BD-I patients who had experienced past psychotic symptoms and a control group. Regarding the self-report of emotions, no evidence was found that both groups differ in their ability to regulate emotions. However, regarding neural measures, the influence of reappraisal on the connectivity from the left DLPFC to the left amygdala was weaker in patients with BD. No significant differences were found for the connectivity of the VLPFC and the amygdala.

Taken together, most studies that investigated reappraisal in euthymic patients reveal no significant behavioral differences in patients with BD and HC (Corbalán et al. 2015; Gruber et al. 2014; Kanske et al. 2015; Kjærstad et al. 2016; Townsend et al. 2013; Zhang et al. 2018). However, one study contrasts with these findings; Rive et al. (2015) did find deficits in the ability to use self-focused reappraisal in euthymic patients with BD-I. Regarding neural measures, amygdala hyperactivity of euthymic and euthymic patients suggests less effective reappraisal (Corbalán et al. 2015; Kanske et al. 2015). Analyses of functional connectivity revealed aberrations in the connectivity between the amygdala and OFC and ACC (Kanske et al. 2015), VLPFC, occipital gyrus, posterior cingulate, right middle frontal gyrus (Townsend et al. 2013), and DLPFC (Zhang et al. 2018).

### Comparing distraction and reappraisal

In three of the studies we identified, participants applied both distraction and reappraisal. Therefore, these studies allow us to compare both strategies with each other.

Regarding behavioral measures, Heissler et al. (2014) neither found significant differences between people at risk for BD and HC in the distraction condition nor the reappraisal condition. Regarding neural measures, participants at risk for BD showed a heightened activity of the amygdala during the reappraisal of negative emotions compared to HC. During distraction from positive emotions, participants at risk for BD exhibited increased activity in the left inferior parietal cortex.

Kanske et al. (2015) found distraction and reappraisal for BD-I patients to be an effective strategy to regulate positive and negative emotions at the behavioral level. However, for people at risk for BD compared to HC, they found reappraisal to be more effective than distraction. Regarding neural measures, neither patients with BD-I nor individuals at risk for BD differed significantly from HC.

Lois et al. (2017) investigated neural network connectivity during reappraisal and distraction in euthymic patients with BD-I and HC. The patient groups showed aberrantly increased intra-network and inter-network connectivity in the default mode network during distraction compared to the reappraisal condition, which indicates that distraction and reappraisal in patients with BD can be distinguished by specific activity patterns in large-scale brain networks.

Taken together, the findings of studies investigating reappraisal and distraction show that the two ER strategies can be distinguished based on their use of large scale networks. Impairments can occur for both strategies in BD. A direct comparison suggests that reappraisal has an advantage over distraction.

## Discussion

Aberrant emotion reactivity (Mercer and Becerra 2013; Rosen and Rich 2010; Townsend and Altshuler 2012; Wessa and Linke 2009) and regulation of emotions are predominant features of bipolar disorder (Aldao et al. 2010; Dodd et al. 2019; Green et al. 2011; Gruber et al. 2013a; Phillips 2003; Phillips et al. 2008; Townsend and Altshuler 2012). The present article reviews studies that examined explicit ER in patients with BD and/or individuals at risk for BD. The compilation of the results leads to a threefold conclusion: First, we highlight the potential role of neural activity during explicit ER as a specific marker indicating the risk for the development of BD. Second, we highlight the role of ventral-rostral regions of the ACC in patients with current affective episodes, and third, we describe possible neural mechanisms that prevent euthymic patients from showing behavioral deficits in ER.

We identified 15 studies investigating explicit ER via maintenance, distraction, situation-focused reappraisal, and self-focused reappraisal of emotions. On a subjective level, self-report measures of valence or arousal were used. Objective measures included the analysis of facial expression and physiological parameters (skin conductivity, respiratory sinus arrhythmia, and MRI). Different patient and risk groups were tested; most diversely, reappraisal was investigated in participants at risk for BD, euthymic patients, and patients in a depressed or hypomanic state. Maintenance of emotions was studied in euthymic patients. Studies on distraction examined participants at risk for BD and euthymic patients. A specific characteristic of the distraction studies is that impairments can manifest in either reduced down-regulation of emotional sensitivity through distraction or in hampered performance in the task that is intended to provide a distraction (e.g., a cognitively demanding task) (van Dillen et al. 2009; Wessa et al. 2013). In the following, we discuss and synthesize these studies along the different participant groups to carve out the potential etiological role of ER deficits in BD.

### The potential role of explicit emotion regulation as a marker for bipolar disorder

Recent research has highlighted the importance of identifying specific markers that indicate the risk of BD for early intervention and prophylactic treatment (Malhi et al. 2017). Risk groups are particularly well suited to identify such markers.

#### Explicit emotion regulation in risk groups

In terms of behavioral measures, the reviewed studies suggest a relatively intact ER of risk groups. Regarding distraction, no impairments were found. Regarding reappraisal, two of three studies that investigated reappraisal in individuals at risk for BD revealed no significant impairments at the behavioral level (Ajaya et al. 2016; Heissler et al. 2014).

In contrast, the third study revealed reduced reappraisal effects in the risk group (Kanske et al. 2015). A crucial difference between these studies is the selection of risk groups. The studies that found no behavioral differences in reappraisal selected participants based on personality traits. In contrast, the study that examined first degree relatives of BD patients showed impaired reappraisal. Because the experimental setup of Kanske et al. (2015) and Heissler et al. (2014) was identical, the different selection of risk groups could cause contradictory findings. However, given that the difference between these risk groups was not systematically investigated, a conclusive interpretation of these findings is difficult. Previous studies have shown that both genetic risk and personality traits are highly predictive of the development of BD (Craddock and Sklar, 2013; Johnson et al. 2015; Kwapil et al. 2000; Rasic et al. 2014; Walsh et al. 2015).

#### Neural deviations in risk groups during explicit emotion regulation

Because risk groups showed sustained ER in most studies, the underlying neural activity of explicit ER is particularly interesting. Regarding distraction, this group exhibited aberrations in neural networks associated with emotion reactivity and ER (Heissler et al. 2014; Ladouceur et al. 2013). Individuals at risk exhibited VLPFC hyperactivity and reduced positive functional connectivity between VLPFC and the amygdala and the DLPFC during distraction from positive emotional stimuli (Ladouceur et al. 2013). During the distraction from positive emotional stimuli in an arithmetic task, individuals at risk showed hyperactivity in the left inferior parietal cortex, an area related to arithmetical operations (Heissler et al. 2014). Taken together with the behavioral results, these findings suggest that while individuals at risk can distract themselves from emotional stimuli at a level comparable to HC, this ability may be associated with increased cognitive effort, as indicated by hyperactivity in the corresponding areas. Please note, however, not all studies found significant neural aberrations in risk groups during distraction (Kanske et al. 2013, 2015). Regarding reappraisal, individuals at personality-based risk show hyperactivity in the amygdala (Heissler et al. 2014), which is also present in first-degree relatives, where aberrant positive functional connectivity between the amygdala and parts of the regulation network were demonstrated (Kanske et al. 2015).

#### Deviations in risk groups: implications for theories on the etiology of bipolar disorders

These findings in risk groups provide information on whether aberrations in emotion reactivity and regulation of individuals with affective disorders are vulnerability factors that exist before the development of a disorder as claimed by the trait marker hypothesis (Rohde et al. 1990), or the consequence of affective episodes that occur during an affective disorder as claimed by the scar hypothesis (Rohde et al. 1990). Concerning these opposing hypotheses, the behavioral findings of the reviewed studies do not provide a definite conclusion. While one study revealed behavioral impairments in the explicit ER of individuals at risk for BD (Kanske et al. 2015), the majority of studies found no significant differences between risk groups and HC regarding behavioral measures (Heissler et al. 2014; Kanske et al. 2013; Ladouceur et al. 2013). However, in contrast to behavioral impairments, most studies revealed aberrations in frontostriatal areas (Heissler et al. 2014; Kanske et al. 2015; Ladouceur et al. 2013). Given that the reviewed studies' risk groups did not experience any preceding affective episodes, aberrations in explicit ER provide evidence for the trait marker hypothesis. These aberrations often cannot be detected in behavioral measures of explicit ER but are initially only visible in more sensitive neural measures. Interestingly, however, in a study that examined individuals at genetic risk, impairments in reappraisal were revealed on the behavioral level. In this sample, the additional activity alterations in areas involved in implicit ER (i.e., OFC and ventral ACC) may have led to additional behavioral impairments, which underlines the role of areas associated with implicit ER in explicit ER.

Concerning the use of explicit ER as a marker for the BD risk, neural aberrations during explicit ER seem to be promising due to their high sensitivity relative to behavioral measures. Most importantly, especially the regulation of positive emotions seems to be associated with neural aberrations (Heissler et al. 2014; Ladouceur et al. 2013). This detail is essential because valence-specific aberrations potentially distinguish bipolar disorders from other affective disorders, especially depression. Recently, it has been shown that patients with BD and UD can be distinguished based on structural and (valence-specific) functional neural aberrations associated with altered ER. (Bürger et al. 2017; Redlich et al. 2014; Repple et al. 2017). Concerning prophylactic treatment, in particular, the differentiation during the early course of the disorder could be critical. Although research on explicit ER of positive emotions in patients with major depression disorder and especially in risk groups is relatively rare, there is evidence to suggest that individuals at risk for unipolar depression (UD) differ from individuals at risk for BD regarding neural changes during explicit ER. For example, patients with UD exhibit decreased activity in the VLPFC during explicit ER of positive emotions (Canli et al. 2004; Kanske et al. 2012), which is the opposite of what was found for individuals at risk for BD during distraction from positive emotions (Ladouceur et al. 2013). To our knowledge, only one study investigated neural aberrations in individuals at risk for UD during ER of positive emotions (Simsek et al. 2017). This study revealed no significant aberrations. However, these studies' results cannot be regarded as conclusive, especially as there are, to our knowledge, no studies that have directly compared both risk groups.

Also, longitudinal studies are indispensable to investigate if, in BD risk groups, neural activity in areas associated with implicit and explicit ER is predictive and specific of developing BD later on. Nevertheless, the results in risk groups provide essential information about neural changes underlying BD since the results are not possibly confounded by medication or treatment effects. Studying medicated participants can lead to results that are challenging to interpret because it is difficult to determine whether differences between patients and HC are due to medication, the disorder, or an interaction of both. Additionally, patients—especially euthymic/remitted ones—have mostly undergone some form of psychotherapy that often specifically trains explicit emotion regulation, a factor that should be considered when interpreting the results in patient samples. However, recent tools such as the tracking software chronocord (Bauer et al. 2019; Pilhatsch et al. 2018) can be used to determine mood, sleep, and medication more specifically and can investigate their effects on emotion regulation and neural activity in patient groups.

### The role of the rACC in emotion regulation during affective episodes

The literature search revealed a lack of studies investigating patients with current depressive, manic, and hypomanic episodes.

Only one study investigated reappraisal in BD patients with a depressive episode (Rive et al. 2015). This patient group exhibited valence-specific aberrations during reappraisal. Reappraisal of sad emotions was accompanied by hyperactivity in the rACC, and the reappraisal of happy emotions was associated with decreased activity in this area. In previous studies, the rACC has been associated with resolving emotional conflicts (Etkin et al. 2006). Rive et al. (2015) hypothesize from valence-specific aberrations in the rACC that the reappraisal of mood-congruent emotions for BD patients with a depressive episode represents an emotional conflict. This hypothesis raises whether aberrant activity in the rACC is also evident for patients in a manic or hypomanic episode regulating mood-congruent emotions.

Likewise, only one study that we identified in our literature search investigated hypomanic patients; however, they were part of a mixed sample, including euthymic patients (Morris et al. 2012). Unfortunately, the authors did not investigate positive, that is, mood-congruent emotions for hypomanic patients. However, BD patients exhibited hyperactivity in rACC during the upregulation of negative emotions, but not during downregulation. While these findings do not contrast with the hypothesis of Rive et al. (2015), according to which an emotional conflict arises in the downregulation of emotions corresponding to the current mood, these findings indicate a further emotional conflict in patients with BD, which may contribute to difficulties in the emotional regulation of this patient group.

In sum, the findings of studies that investigated patients with current affective episodes suggest that the downregulation of mood-congruent and the upregulation of mood-incongruent emotions is associated with aberrant activity in the rACC. With reciprocal connections to the DLPFC, VLPFC, other parts of the ACC, and the amygdala, the rACC takes a prominent role within the ER network (Etkin et al. 2006, 2011; Phillips et al. 2008; Tang et al. 2019). The consistent reporting of aberrations in the rACC in BD patients with affective episodes suggests that this area is of particular relevance for the deficit ER of BD patients. Interestingly, aberrant activity in the rACC is also a neural differentiator between individuals at risk for BD and patients with current episodes. While both groups exhibit hyperactivity in the amygdala and the VLPFC and aberrant functional connectivity between these areas (Heissler et al. 2014; Kanske et al. 2015; Ladouceur et al. 2013), hyperactivity in the rACC seems to be exclusive for patients with current affective episodes (Morris et al. 2012; Rive et al. 2015). Revisiting the scar hypothesis, an interesting question is whether this aberrant rACC activity can also be found in remitted/euthymic patients. Since no aberrations were found in the rACC of risk groups, this would indicate that the aberrant rACC activity is more a consequence of the disorder than a risk factor, which would provide evidence for the scar hypothesis.

### Impairments of explicit emotion regulation in euthymic individuals

As outlined above, impairments in distraction can manifest in two ways. First, in impairments in the cognitive distraction task, and second in the experience of emotions as measured via self-report. Therefore, the results for distraction in euthymic BD patients are challenging to interpret. While in euthymic patients, measures concerning the distraction task's performance suggest impaired distraction (Caseras et al. 2015; Kanske et al. 2013), self-report measures of emotions revealed no significant differences between euthymic patients and HC (Kanske et al. 2015). Thus, taken together, the results suggest that euthymic patients may regulate their emotions successfully but at the expense of performance in cognitive tasks. In other words, distraction may require a higher cognitive effort in euthymic patients.

Regarding behavioral measures of reappraisal, the majority of studies did not find evidence for deficits in euthymic patients (Corbalán et al. 2015; Gruber et al. 2014; Kanske et al. 2015; Kjærstad et al. 2016; Townsend et al. 2013; Zhang et al. 2018). The only study that revealed impaired reappraisal at the behavioral level differs from the other studies in that self-focused reappraisal was used instead of situation-focused reappraisal (Rive et al. 2015). Regarding the explicit ER strategy of maintaining emotions (Gruber et al. 2013a), euthymic patients with BD-I showed reduced maintenance of negative emotions. No impairments were found for positive emotions. Phases of extensively elevated positive mood (= mania) are one core characteristic of BD. The results imply that patients with BD experience such phases because negative emotions that otherwise suppress positive emotions are maintained deficiently. In contrast to patients with BD, patients with UD were as able as HC in maintaining negative emotions (Gruber et al. 2013a, b). By explaining elevated mood in BD by a deficit in maintaining negative emotions, the authors thus provide an interesting etiological explanation for manic phases in patients with BD that distinguishes BD from other affective disorders. Further research is needed to provide a solution for inconsistencies that arise from this approach. For example, it does not explain depressive episodes in BD or that BD patients rate positive emotions less positively (Kanske et al. 2015). Further research with patients in different mood states could help resolve these inconsistencies.

In sum, the reviewed studies descriptively suggest differences in the effectiveness of different explicit ER strategies for euthymic patients. While the results indicate that (situation-focused) reappraisal may be an effective strategy for euthymic patients, due to increased cognitive effort during distraction in patients with BD, distraction seems less effective. On the other hand, studies systematically comparing distraction and reappraisal in euthymic patients did not find significant differences in both strategies' effectiveness (Kanske et al. 2015; Lois et al. 2017). Given further research efforts in this field, future studies may use meta-analytical methods to clarify differences in both strategies in euthymic patients.

#### Neural deviations in euthymic patients during explicit emotion regulation

Regarding neural measures, structural and functional aberrations in euthymic patients with BD were revealed (Caseras et al. 2015). During distraction, this group exhibited hyperactivity in DLPFC, amygdala, and nucleus accumbens. Interestingly, these findings differ between patients with BD-I and patients with BD-II. While the BD-I group exhibited hyperactivity in the areas mentioned above during distraction from happiness, the BD-II group exhibited the most pronounced aberrations during distraction from fear. Unlike BD-I patients, BD-II patients also exhibited increased inverse functional connectivity between DLPFC and amygdala during the distraction from fear. This finding is particularly interesting, combined with the behavioral findings of BD-II patients. Behaviorally, patients with BD-II showed no differences in the distraction of fear distractors compared to HC, which suggests that the strengthened connection between DLPFC and amygdala may be a compensatory mechanism that prevents behavioral losses.

Another study did not find any significant differences between euthymic BD patients and HC on the neural level (Kanske et al. 2015). However, again taking into account findings of hyperactivity in the right parietal cortex of patients with BD-I during distraction with an arithmetic task, a different picture emerges (Kanske et al. 2013). The right parietal cortex is associated with arithmetic tasks (Park et al. 2013). In line with the behavioral results, both studies suggest that BD patients can distract from emotions, but only by investing more resources in the distraction task.

Regarding reappraisal, hyperactivity in the amygdala of euthymic patients indicates impairments in ER (Corbalán et al. 2015; Kanske et al. 2015). Further, the reviewed studies revealed aberrations in frontal areas' functional connectivity and the amygdala (Kanske et al. 2015; Townsend et al. 2013; Zhang et al. 2018). However, the results are ambiguous as to which frontal areas are involved. Aberrant connectivity to the amygdala was found for the VLPFC, occipital gyrus, right posterior cingulate, right middle frontal gyrus (Townsend et al. 2013), DLFPC (Zhang et al. 2018), OFC, and ventral ACC (Kanske et al. 2015). While VLPFC and DLPFC were previously associated with explicit ER, the OFC and ventral ACC was more likely to be associated with implicit ER (Etkin et al. 2015; Phillips et al. 2008). Of particular interest here is the aberrant activity in the ventral ACC. Given that the ventral ACC and rACC together build a subsystem of the rACC (Etkin et al. 2011), as described above, aberrant activity in the rACC of euthymic patients is evidence for the scar hypothesis, which claims that the experience of affective episodes leads to permanent changes in patients with affective disorder. An interesting pending question is whether changes in activity in the ventral ACC predict the likelihood of recurrence.

#### Discrepancies between behavioral and neural measures in euthymic patients

A remarkable result of the studies investigating distraction in euthymic patients is the discrepancy between behavioral and neural results. While especially the studies that investigated situation-focused reappraisal did not find any significant behavioral differences between euthymic BD patients and HC (Corbalán et al. 2015; Gruber et al. 2014; Kanske et al. 2015; Kjærstad et al. 2016; Townsend et al. 2013; Zhang et al. 2018), neural aberrations in the amygdala, frontal areas, as well as in the functional connectivity between these areas were found. Regarding distraction, the studies reviewed suggest that euthymic patients with BD can successfully distract themselves from emotions, but this ability is associated with increased connectivity between areas associated with cognitive control and the amygdala (Caseras et al. 2015).

One possible explanation for the discrepancy is that it may be a statistical artifact resulting from underpowered study designs. (see the Limitations section of this article). An alternative explanation may be that neural aberrations represent compensatory mechanisms that prevent impairments on the behavioral level (Kjærstad et al. 2016). Considering that models of the neural basis of ER assume that frontal areas associated with cognitive control act to regulate striatal areas of emotion reactivity, this explanation also seems plausible. Aberrations were found in frontal areas and the connectivity between frontal and striatal areas. Further indications of a compensatory mechanism can be inferred from studies in which euthymic patients showed increased recruitment of areas associated with the distraction task but did not differ significantly from HC in terms of perceived emotions (Kanske et al. 2013, 2015). Further plausibility for the compensation hypothesis arises from the fact that most euthymic patients examined in the reviewed studies were on medication and/or psychotherapeutic treatment.

Given that medication and psychotherapy have compensatory effects on symptoms of mental disorders, it seems plausible if compensatory mechanisms are also reflected in brain activity. In any case, the interpretation of neural changes in euthymic patients must consider the effects of medication and psychotherapy. The next section outlines the implications of the findings for psychotherapy and trainability in more detail.

### Implications

The studies that examined explicit ER in patients with BD showed aberrations in areas associated with explicit ER (e.g., DLPFC and VLPFC) and areas associated with implicit ER (e.g., OFC and ACC). These findings are also consistent with the model of Phillips et al. (2008); it assumes a partially joint route of explicit and implicit ER. This joint route is particularly interesting in light of studies showing that patients with BD exhibit deficits in using effective implicit ER in their daily lives. If activating the explicit route also activates the implicit route, this has important implications for trainability. Frequent (co-) activation of the implicit route when using explicit ER might also lead to implicit ER improvements. Since explicit ER can be trained in a controlled manner, it could also improve implicit ER.

Indeed, previous studies reveal evidence for the trainability of ER (Schweizer et al. 2013). Training with an emotional working memory paradigm affects neural activity in areas associated with explicit emotion regulation (like the DLPFC and VLPFC) and areas more associated with implicit emotion regulation (like the OFC and ACC) as well as the connectivity between them.

Another approach to improving ER in BD patients is to train patients to use ER strategies that are most effective for them, as studies have shown the importance of choosing an appropriate ER strategy for successful ER (Sheppes et al. 2014). This point is particularly relevant in light of the reviewed studies that have directly compared different ER strategies. On the one hand, these studies can show whether and how BD patients can fall back on successful ER strategies and, on the other hand, whether there are deficits that still need to be addressed. Given that it is relatively spared in BD, distraction may be a resource that BD patients can exploit, while they may not benefit from applying reappraisal. On the other hand, patients could benefit from more frequent reappraisal use in the long run because repeated use could act like training and lead to a more successful application. The frequently found aberrations in fronto-limbic connectivity during reappraisal in BD may indicate precisely this potential for improvement. Moreover, as healthy individuals at risk for BD show similar fronto-limbic abnormalities, training reappraisal may also function as a preventive intervention.

In summary, our review suggests the following conclusions. First, individuals at risk for BD exhibit neural aberrations, which may be a specific marker to distinguish the risk of BD from the risk of unipolar depression. Secondly, individuals at risk for BD and euthymic patients may compensate for ER deficits by enhanced recruitment of structures related to cognitive control and/or relevant for the distraction task. Our review found that both distraction and reappraisal are effective strategies for BD patients and individuals at risk for BD to regulate their emotions.

### Limitations

The present review aimed to apply a strict definition of explicit ER to describe specific neural and behavioral aberrations in explicit ER in individuals at risk for BD, patients with a current affective episode, and euthymic patients. We further classified the reviewed studies based on the ER strategy investigated (i.e., reappraisal, distraction, and maintenance). These classifications entail limitations because, regarding specific samples in combination with specific ER strategies, only a few studies are available. Further, it should be noted that not all ER strategies are equally represented in research. Most studies investigated reappraisal, followed by distraction, and last, maintenance. Similarly, in terms of mood state, most studies investigated euthymic patients. Only two studies investigated patients with an affective episode.

Although these classifications lead to less robust conclusions due to the small number of studies in each class, they have the advantage of allowing a more fine-grained description of the variations in ER of individuals with or at risk for BD.

Furthermore, the conclusions on explicit ER as a marker for BD are subject to limitations. On the one hand, the reviewed studies show that altered explicit ER is present in risk groups, primarily evidenced by altered neural processing; on the other hand, it is unclear how specific these alterations are in differentiating them from other disorders with alterations in emotion regulation. While we addressed the differences between patients with BD and unipolar depression in discussing the results, we did not include other disorders in which ER disorders are present (e.g., borderline personality disorder and schizophrenia). This selection is due to the lack of studies comparing these groups. In particular, it should be emphasized here that the known markers according to which risk groups can be determined cannot themselves specifically distinguish between different affective disorders. For example, the first-degree relatives of BD patients who constitute the risk group in some of the reviewed studies show not only an increased risk of developing BD themselves but also an increased vulnerability to other disorders (Rasic et al. 2014). Longitudinal studies are needed that allow classification into specific risk groups after the onset of the disorder and assess explicit ER before the onset.

Many of the reviewed studies reported null findings, making it difficult to reach a conclusive estimate of intact or impaired ER in individuals with BD or at risk for BD. On the one hand, this is due to the theoretical conception of frequentist significance tests, and on the other hand, many neuroscientific studies are underpowered (Poldrack 2017). Reporting statistical power in frequentist statistics or a switch to bayesian hypothesis tests with planned precision would allow a more precise interpretation of the results (Kruschke and Liddell 2018). All of the reviewed studies used frequentist significance tests. Most of them did not report statistical power that would allow more accurate handling of any null results. Accordingly, caution is advised in our synthesis and interpretation of the findings. In future research, meta-analytic approaches may draw more accurate conclusions.

Another difficulty concerning the interpretation of the present studies arises from the medication of the samples studied. Only a single study, which examined patients with BD, investigated a medication-free patient sample. Although studying medicated samples can provide important insights, it is essential for causal research of BD to increasingly control for medication. Similar concerns apply to research on patients in euthymic mood state. On the one hand, research with remitted patients provides essential insights, particularly concerning the consequences of affective episodes. On the other hand, it reveals little about the causes of BD, as any differences between remitted patients and HC may result from medication, psychotherapy, or change in life circumstances related to the disorder. Without adequately accounting for these factors, it remains difficult to separate them in interpretation.

In recent research, different response styles of emotion regulation have been increasingly studied (Nolen-Hoeksema 2004). The term response styles describe the individual tendency to react to emotions in a certain way (e.g., a tendency to ruminate). Since this tendency is rather implicit, it is outside the scope of the present review. This exclusion is a possible limitation of the present review, as the explicit regulation of different response styles could represent an interesting interaction between explicit and implicit ER. On the other hand, this would contradict the strict definition of explicit ER that we based the review on and further complicate the already difficult distinction between explicit and implicit ER at the neural level. For this reason, we decided against including research on response styles.

## Data Availability

Not applicable.
